# Presenting national HIV/AIDS and sexually transmitted disease research in Brazil

**DOI:** 10.1097/MD.0000000000010109

**Published:** 2018-05-25

**Authors:** Adele Schwartz Benzaken, Maria Cristina Pimenta Oliveira, Gerson Fernando Mendes Pereira, Silvana Pereira Giozza, Flavia Moreno Alves de Souza, Alessandro Ricardo Caruso da Cunha, Renato Girade

**Affiliations:** Department of Surveillance, Prevention and Control of STIs, HIV/AIDS and Viral Hepatitis, Ministry of Health, Brasília, Brazil.

In 2016, an estimated 830,000 individuals were living with HIV/AIDS in Brazil, with an overall HIV prevalence rate of 0.4% in the general population. In the same period, 37.4% of AIDS cases occurred among young people aged 20 to 29. The male/female sex ratio of AIDS cases increased from 1.8 in 2010 to 2.5 in 2016. Among men, 60% of the 2016 cases occurred among men who have sex with men (MSM).^[1]^ Thus, the HIV epidemic in Brazil remains a concentrated epidemic with prevalence above 5% in specific populations, such as MSM, transgender women, female sex workers, and people who use drugs. The National Department of Surveillance, Prevention and Control of sexually transmitted diseases (STIs), HIV/AIDS and Viral Hepatitis has funded new epidemiological and behavioral surveillance surveys to monitor the HIV, syphilis, and hepatitis B and C epidemics in specific vulnerable populations, and young men enlisted as military conscripts. Initial prevalence results are presented in this supplement.

In December 2017, epidemiological data for the country also indicated a reduction trend in the number of AIDS cases and mortality caused by AIDS in Brazil. In recent years, care cascades have been used as important tools to guide decision-making and the design of health policies based on qualified information. Likewise, through them, the progress and results of the efforts undertaken to reach the 90–90–90 goals proposed by Joint United Nations Programme on HIV/AIDS for 2020, also adopted by Brazil, may be analyzed considering the estimated number of persons living with HIV (PLHIV) in the country.^[2]^

Cascade monitoring has demonstrated the assertiveness of the treatment and care policies, which have increased HIV diagnosis, shortened the time to start treatment, and, consequently, increased the number of people receiving antiretroviral therapy.^[3]^

Brazil's public health structure is based on a Unified Health System (SUS), created under the 1988 Federal Constitution when access to integral health care became a universal right to all citizens and a duty of the State. SUS is a decentralized system with articulated services and programmatic actions in all 3 levels of the Federation: Federal, States, and Municipalities. The national HIV response is built upon this system.

The Brazilian national HIV response has been effectively developed for 3 decades under the coordination of the National Department of Surveillance, Prevention and Control of Sexually Transmitted Infections, HIV/AIDS and Viral Hepatitis of the Ministry of Health, based on scientific evidence and policy innovation, as a result of the political will of committed policy makers, a dedicated scientific community, an engaged civil society, and also crucial national, regional, and international partnerships.

During the past decade, the HIV national response has expanded the populations’ access to rapid HIV testing and treatment access with the aim of improving the quality of life of those living with HIV and containing epidemic expansion, given the transmission obstructing effect of ART, as demonstrated by scientific evidence in 2011.^[4–6]^ In December, 2013, Brazil took another bold step by becoming the first upper middle-income country to establish a policy of treating all regardless of their CD4 count.^[7–10]^ This policy was rolled out in these last few years. At present, the recently approved national policy for primary HIV prevention will make pre-exposure prophylaxis (PrEP) available to key populations at greater risk of HIV infection, using a combination prevention strategy as an alternative functional tool for HIV prevention.

Along the years, extensive programmatic efforts have been made to continuously measure the public health impact of these initiatives on HIV transmission and monitor improvements in the quality of life of those PLHIV and AIDS, including markers related to stigma and discrimination against specific populations. Underlining this effort is the continuous responsibility and dedication of government officials in partnership with the scientific community, respected research institutes, and the support of civil society, to generate knowledge for country-specific programmatic decision-making in a public health context of the dynamics of the AIDS epidemics in a country with many political and economic challenges to overcome.

The articles presented in this special supplement intend to address some of the knowledge gaps identified in the context of the programmatic ventures faced in Brazil under the current AIDS epidemic, and also contribute with knowledge dissemination, particularly to those countries with similar concentrated epidemics.

This special supplement presents recent research production supported by the National HIV/AIDS Department, with the objective of generating crucial scientific knowledge to inform public health policies. Manuscripts considered for inclusion were chosen from papers submitted by investigators with extensive publishing experience in international peer-reviewed journals and specially invited to participate in the selection process.

A total of 15 manuscripts were submitted and 11 accepted for publishing as part of this special supplement, providing a sample of current and relevant research studies and methods applied in Brazil, ranging from epidemiological and behavioural studies with specific key populations to laboratory science. A primary purpose of these studies is to provide scientific knowledge to inform prevention and treatment of STIs, HIV, and viral hepatitis public health policies, support vital decision-making, and guide programmatic implementation.

This Special Supplement includes: 6 articles derived from national HIV, HCV, HBV, and syphilis biological and behavioral surveillance surveys conducted in 2016 to 2017, in 12 cities of 5 macro-regions of Brazil, using respondent-driven sampling (RDS) methodologies in MSM populations, female sex workers, and transgender women; an article on external quality assessment for CD4+ T-lymphocyte count tests of the national laboratory network; an article on national HIV prevalence and sexual behavior of young male conscripts; a national cross-sectional study to monitor self-reported adherence to antiretroviral therapy in public HIV care facilities in Brazil; an article on de demand of PrEP for key populations; and n article on social and demographic determinants of attrition in the HIV continuum of care in Brazil.

Therefore, the articles presented in this supplement are not only of national interest and importance, but contribute with global thinking and knowledge dissemination so crucial to invigorate surveillance, prevention and care, and confront dynamic epidemics under a public health perspective.

## Acknowledgment

We would like to acknowledge UNESCO's support in the production of this supplement.

## Author contributions

**Conceptualization:** Maria Cristina Pimenta Oliveira.

**Project administration:** Renato Girade.

**Resources:** Adele Schwartz Benzaken.

**Supervision:** Adele Schwartz Benzaken.

**Validation:** Adele Schwartz Benzaken, Gerson Fernando Mendes Pereira.

**Writing – original draft:** Maria Cristina Pimenta Oliveira.

**Writing – review & editing:** Maria Cristina Pimenta Oliveira, Gerson Fernando Mendes Pereira, Flavia Moreno Alves de Souza, Silvana Pereira Giozza, Alessandro Ricardo Caruso da Cunha.
